# Hybrid RNA/DNA Concatemers and Self-Limited Complexes: Structure and Prospects for Therapeutic Applications

**DOI:** 10.3390/molecules29245896

**Published:** 2024-12-13

**Authors:** Maria A. Kanarskaya, Sofia V. Novikova, Alexander A. Lomzov

**Affiliations:** 1 Institute of Chemical Biology and Fundamental Medicine SB RAS, Novosibirsk 630090, Russia; makanarskaya@gmail.com (M.A.K.); s.kosvintseva@g.nsu.ru (S.V.N.); 2 Department of Physics, Novosibirsk State University, Novosibirsk 630090, Russia

**Keywords:** hybrid RNA/DNA complex, self-limited complex, concatemer, rational design, self-assembly, nucleic acid supramolecular complex, nucleic acid structure

## Abstract

The development of new convenient tools for the design of multicomponent nucleic acid (NA) complexes is one of the challenges in biomedicine and NA nanotechnology. In this paper, we analyzed the formation of hybrid RNA/DNA concatemers and self-limited complexes by a pair of oligonucleotides using UV melting, circular dichroism spectroscopy, and a gel shift assay. Effects of the size of the linker between duplex-forming segments of the oligonucleotides on complexes’ shape and number of subunits were compared and systematized for RNA/DNA, DNA/DNA, and RNA/RNA assemblies. The data on complex types summarized here as heat maps offer a convenient tool for the design of NA constructs. General rules found for RNA/DNA, DNA/DNA, and RNA/RNA complexes allow not only designing complexes with desired structures but also purposefully transforming their geometry. The A-form of the double helix of the studied RNA/DNA complexes was confirmed by circular dichroism analysis. Moreover, we show for the first time efficient degradation of RNA in hybrid self-limited complexes by RNase H and imidazole. The results open up new prospects for the design of supramolecular complexes as tools for nanotechnology, nanomachinery, and biomedical applications.

## 1. Introduction

In the last 20 years, major developments have been carried out in the field of nucleic acid (NA) nanotechnology and nanomedicine [1,2,3,4,5,6,7,8,9]. The creation of nanostructures based on oligonucleotides is one of the most attractive areas of science and technology [1,2,3,4,5,6,7,8,9]. NA-based structures have high potential in various applications in the fields of biomedicine [1,2,3,4,5,6,7] and nanotechnology [8,9]. Nanoconstructs, on the one hand, can be used for molecular therapy [1,2,3,4,5,6,7] and diagnostics [4,5,6], or, on the other hand, as molecular transporters, shuttles, walking robots, pumps, logic gates, amplifiers, and other devices [8,9].

RNA/DNA hybrid supramolecular complexes are of interest [10,11,12,13,14,15]. These structures can regulate gene expression, participate in various biological processes, and serve as carriers for therapeutic nucleic acids (TNAs) [10,13]. In addition, an oligonucleotide can self-associate into complexes with a TNA, and these complexes can regulate biological processes [3,10,14,15,16]. For example, a target RNA (e.g., mRNA or microRNA) in a hybrid complex with DNA can be digested by RNase H or artificial ribonuclease (aRNase; e.g., [3,17]). For these purposes, RNA-containing nanoassemblies can be rationally designed, optimized, and engineered for specialized in vivo applications. Many one-dimensional (1D), 2D, and 3D constructs with unique structural attributes of RNA, DNA, or RNA/DNA hybrids have been developed and studied. A number of recent reviews summarized the advances in this research area [4,9,14,18,19,20,21]. Extensive studies have been conducted on the safety and immunological and pharmacological profiles of RNA-containing nanoparticles and have paved the way for clinical trials [4,5,21,22,23]. These articles show that RNA nanotechnology holds significant promise for cancer treatment.

**Figure 1:**
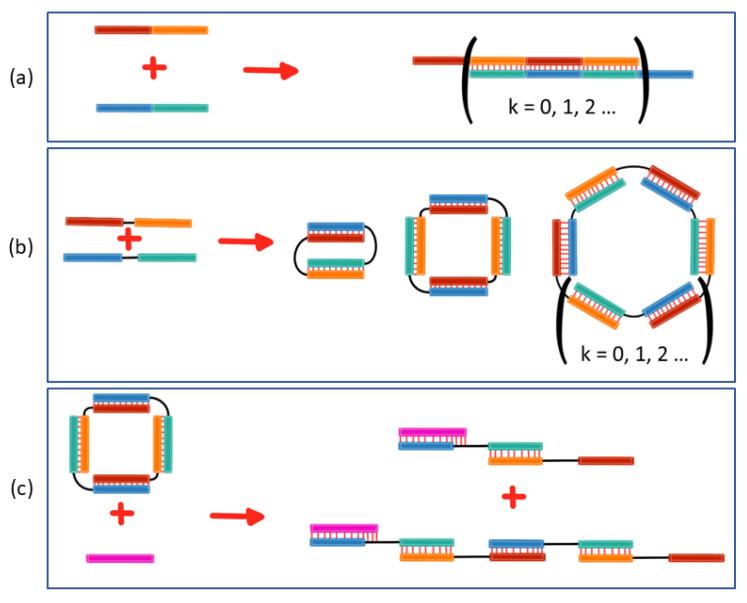
Schemes of the complexes′ formation. (**a**) A concatemer complex; (**b**) self-limited complexes with different numbers of oligonucleotides; (**c**) linearization of a self-limited tetramolecular complex by the addition of an opener (O, pink). The red segment is complementary to the blue segment; the orange segment is complementary to the green segment. Linkers are depicted as black lines. Thin vertical lines denote base pairing.

The diversity of already developed types of NA-based nanostructures is high. Usually, well-studied elements of NA secondary structures, such as duplexes, single-stranded segments, bulges, and loops, are used as building blocks in the nanostructures [11]. The design, synthesis, and assembly of these supramolecular constructs typically require sophisticated techniques and resources. Recently, we devised an approach to the design of self-organizing complexes of various programmable geometries, formed in a controlled manner by only two oligonucleotides [24,25]. Two DNA or RNA oligomers containing a pair of complementary segments or sticky ends can form concatemer complexes (Figure 1). The insertion of a non-nucleotide or nucleotide linker between duplex-forming segments leads to the formation of self-limited complexes of various sizes and shapes. The number of subunits in a complex (hereafter: molecularity) and the geometry of the complex depend on both the sizes of the sticky ends and the size and nature of the linkers in the oligonucleotides. One, two, or more oligonucleotides can form such complexes [24,25,26,27]. Due to the differences in properties between DNA and RNA (e.g., thermodynamic stability, geometry of the double helix, and flexibility), strands with similar nucleotide sequences can form complexes of different types and sizes [24,25]. The high potential of such complexes for RNA and DNA nanotechnology and biomedicine is due to their structural simplicity and easy design, synthesis, and assembly, which make them inexpensive to create and use.

Here, we investigated the formation of RNA/DNA concatemers and self-limited complexes by a pair of oligonucleotides. Types and the molecularity of complexes formed by DNA/DNA, RNA/RNA, or RNA/DNA strands were compared. We analyzed the digestion of RNA by RNase H and imidazole to evaluate the possible usefulness of such a self-assembled complex as a TNA element. Thus, the results indicate an ability to design complexes having a desired shape and size. RNA strands in these complexes can be digested by RNase H or by imidazole.

## 2. Results and Discussion

### 2.1. Model Oligonucleotides

To examine the properties of RNA/DNA complexes, we selected oligonucleotides forming two duplex regions of ten base pairs (bp) in length, similar to the oligomers used in studies on properties of DNA/DNA [24] and RNA/RNA complexes [25]. A set of RNA and DNA oligonucleotides RM-Ui (r [5′-CUAACUAACG (U)_i_ CCAUCAUAUG-3′]) and DN-Tj (d[5′-CGTTAGTTAG (T)_j_CATATGATGG-3′]) containing two 10 nt duplex-forming segments and different linkers (i = 0, 1, 2, 3, 5, 7, or 10 and j = 0, 1, 3, 15, or 25) was synthesized and purified (Table S1 in the Supplementary Materials). In addition, oligonucleotide-opener O (r[5′-AAAAACGUUAGUUAG-3′]) was employed to elucidate the molecularity of self-limited complexes. Fluorescein (FAM)-labeled RM series oligonucleotides were used to quantitate RNA digestion (Table S1).

### 2.2. UV Melting Analysis

The type, shape, and molecularity of complexes formed by a pair of oligonucleotides are temperature dependent [24,28,29]. The thermal stability analysis of RNA/DNA complexes was performed to select experimental conditions to determine the type and molecularity of hybrid complexes and analyze the RNA digestion. An equimolar mixture of oligomers RM-Ui and DN-Tj was subjected to the UV melting analysis. Melting temperature (T_m_) was determined as a first-derivative maximum of the UV melting curve. T_m_ was chosen to characterize thermal stability because quantitative analysis of UV melting curves is problematic [24]. Hysteresis was observed between the heating and cooling curves of RNA/DNA complexes (Figure S1). Previously, similar behavior has been documented for complexes formed by two oligoribonucleotides [25]. This effect is due to the hydrolysis of RNA in the presence of Mg^2+^ ions at high temperatures. Therefore, here, similar to our previous work [24], we utilized only heating curves for the UV melting analysis.

The denaturation curves of all RNA/DNA complexes had a single S-shape transition, which corresponded to a single maximum on the differential melting curves (Figure S2). T_m_ values of all investigated complexes are presented in Table 1.

The melting temperatures were found to range from 36.8 to 49.4 °C. Samples without a linker (RM/DN-Tj, j = 1, 2, 3, 15, or 25) have the highest T_m_ values (43.5–49.4 °C). Furthermore, thermal stability proved to be 2.7–7.5 degrees higher than that of similar samples with linkers in oligomers of the RN series. Complexes formed by DNA without a linker and RNA with a nucleotide linker have T_m_ values of 40.6–43.7 °C. The introduction of linker Tj into the DNA strand caused a ≤4.9 degree decrease in the stability of RM-Ui/DN-Tj (i = 1, 2, 3, 5, 7, or 10) for j = 1, 2 and 3, or, in some cases, stabilization by up to 2 degrees for j = 15 and 25.

The examination of the differential melting curves of various complexes showed that the peak of the curves for the samples without a linker in the RM strand is higher and narrower than that of the others (Figure S2). This behavior can be explained by additional stabilization of the complexes owing to a stacking interaction in the nick. A similar shape of melting curves has been found previously for DNA or RNA concatemers [24,25]. Therefore, we can assume the formation of the same complex type for the hybrid RM/DN-Tj complexes.

Thus, we demonstrated that complexes formed by oligonucleotides with different linker lengths are characterized by different melting temperatures (Table 1). Nonetheless, no strict correlation between thermal stability and linker length was observed in the tested series of the complexes.

A comparative analysis of thermal stability between hybrid RNA/DNA and either RNA/RNA or DNA/DNA complexes yielded results consistent with known data on duplexes [30,31,32]. The melting temperature of RNA/RNA complexes ranges from 50 to 60 °C [24], which is higher than that of the RNA/DNA complexes studied here. In both cases, the linker absence in one of the strands gave rise to thermodynamically stabler complexes. The thermal stability of DNA/DNA complexes turned out to be comparable to that of RNA/DNA but somewhat lower.

The same thermal stability data were obtained for the FAM-labeled RNA/DNA complexes (Figure S3 and Table S2). Introduction of the FAM label into RNA resulted in a slight T_m_ change by −1.5 ± 3.0 °C (mean ± standard deviation; Table S3).

The thermal stability analysis of the RNA/DNA complexes (Table 1 and Table S2) revealed that the complexes are completely formed at 25 °C or lower. Therefore, we selected 5 °C to further investigate the type and molecularity of the complexes by a gel shift assay and to evaluate RNA digestion at 25 or 30 °C.

### 2.3. A Gel Shift Assay

**Figure 2:**
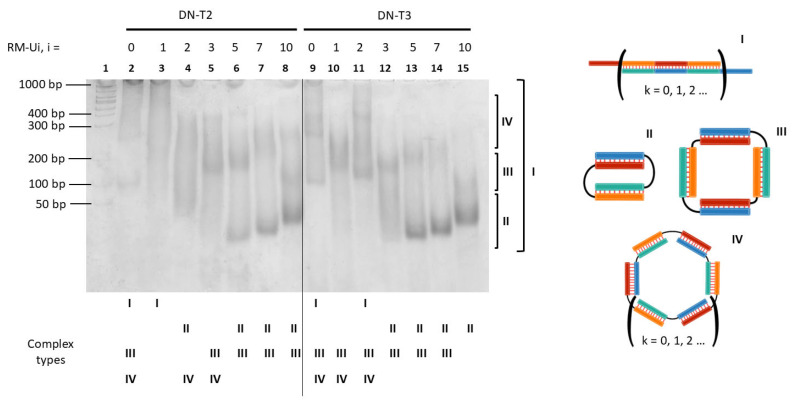
The gel shift assay of oligonucleotides′ complexes RM-Ui/DN-T2 and RM-Ui/DN-T3 with different lengths of linkers (i = 0, 1, 2, 3, 5, 7, or 10). Lane 1: dsDNA ladder of 50–1000 bp; 2: RM/DN-T2; 3: RM-U1/DN-T2; 4: RM-U2/DN-T2; 5: RM-U3/DN-T2, 6: RM-U5/DN-T2; 7: RM-U7/DN-T2; 8: RM-U10/DN-T2; 9: RM/DN-T3; 10: RM-U1/DN-T3; 11: RM-U2/DN-T3; 12: RM-U3/DN-T3, 13: RM-U5/DN-T3; 14: RM-U7/DN-T3; 15: RM-U10/DN-T3. Sizes of bands in the dsDNA ladder (50–1000 bp) are shown on the left, and the correspondence of the bands to the types of complexes is presented on the right of the electropherogram. The types of complexes are indicated by Roman numerals below the electropherogram for each band. Schematic representations of different types of oligonucleotide complexes and their names are given on the right.

This analysis of the hybrid RNA/DNA complexes was performed to determine their type and molecularity. Complexes formed by oligonucleotides with different linkers, RM-Ui/DN-Tj (i = 0, 1, 2, 3, 5, 7, or 10; j = 0, 1, 2, 3, 15, or 25), were examined. Mobility in the gel was determined using a double-stranded (ds) DNA ladder to compare our results with data on DNA and RNA complexes studied before [24,25]. A typical electropherogram is displayed in Figure 2. Complexes with various linkers in oligonucleotides yielded diverse sets of bands. The complexes that gave a band smeared along the lane were assumed to be concatemers (for example, Figure 2, lanes 2 and 3, complex I). A similar observation was made about the DNA/DNA [24,28] and RNA/RNA complexes [25].

Most of the lanes in the electropherograms (Figure 2 and Figures S4–S6) had one or more clear-cut bands that corresponded to self-limited complexes. The mobility of these complexes was in one of three ranges: 35–140, 170–260, or ≥300 bp. We summarized the mobility of all the tested complexes in Tables S4–S6. A similar classification has been proposed for RNA/RNA [25] and DNA/DNA [24] analogs. Based on previously obtained data, it can be hypothesized that the samples with the highest mobility (up to 170 bp) represent complexes containing two oligonucleotides or a bimolecular complex (Figure 2, complex II), the samples with lower mobility (170–260 bp) are a tetramolecular complex (four oligonucleotides, Figure 2, complex III), and the slowest complexes (over 300 bp) have the highest molecularity, i.e., contain six or more oligonucleotides (Figure 2, complex VI). To verify the proposed molecularity of the RNA/DNA complexes, we conducted an additional series of experiments with the gel shift assay in the presence of opener O. An interaction of the opener with self-limited complexes leads to their linearization via the strand displacement mechanism (Figure 1c) [24]. The self-limited complexes consisting of 2 × *k* oligonucleotide strands are restructured into linear complexes of different sizes: 2 × *k* + 1,…, 2 × *k* + 1 − 2 × *m*,…, 3, where *m* is in the range from 1 to *k* − 2 [23]. For instance, when the opener is added to a tetramolecular complex, two additional linearized structures arise, whereas when a bimolecular complex is unfolded, only one additional trimolecular linear complex emerges.

The linearized structures were found to have different sizes and lengths and, consequently, different mobility values in the gel. Thus, the analysis of the additional bands in the lanes after the introduction of the opener at various concentrations allowed us to determine the molecularity of a complex, according to Ref. [24]. Linearization occurs if the interaction of an oligonucleotide contained in a complex with the opener is thermodynamically more favorable than the oligo’s interaction (s) within the complex.

During the design of the opener, an RNA sequence complementary to one of the RNA duplex-forming sites was chosen (Table S1). An RNA opener is preferable to a DNA opener because the thermal stability of RNA/RNA complexes is higher than that of RNA/DNA complexes [30,32]. Extending the opener by five adenines at the 5′ end to interact with the Ui linker should result in additional stabilization of the O/RM-Ui complex. On the other hand, this approach is expected to lead to an accelerated interaction of the opener with self-limited complexes.

**Figure 3:**
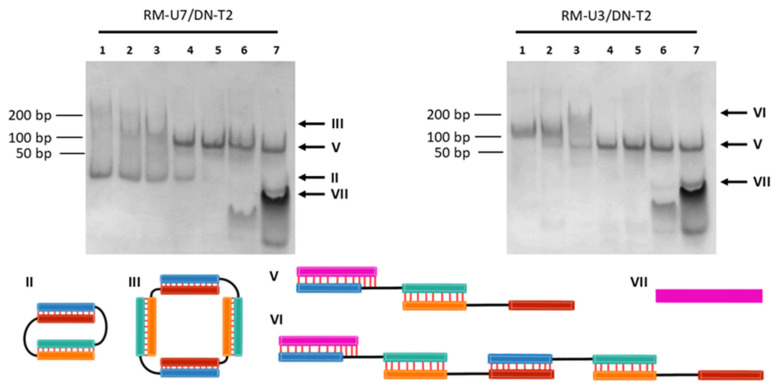
Validation of the presumed molecularity of self-limited complexes by means of their interaction with the opener (O). Gel shift assays of complexes RM-U7/DN-T2 (left) and RM-U3/DN-T2 (right) in the presence of RNA opener O. Ratios of oligonucleotide concentrations [RM-Ui]:[DN-Tj]:[O] in the lanes: 1, 1:1:0; 2, 1:1:0.25; 3, 1:1:0.5; 4, 1:1:0.75; 5, 1:1:1; and 6, 1:1:2; 7, 1:1:10; a value of 1 denotes 10 μM. The types of complexes are presented on the right of electropherograms by Roman numerals. A schematic representation of types of oligonucleotide complexes and their names are shown below. The color codes of oligonucleotides and complexes are identical to those in Figure 1. A dsDNA ladder of 50–1000 bp is shown on the left.

To verify the proposed molecularity, two complexes, RM-U3/DN-T2 and RM-U7/DN-T2, were selected, which have mobility characteristics of bi- and tetramolecular complexes. The opener was added to a solution of a complex at different concentrations at the following ratios [O]:[RNA complex]: 0, 0.25, 0.50, 0.75, 1.00, 2.00, or 10.00. The addition of the opener to RM-U3/DN-T2 or RM-U7/DN-T2 induced a redistribution of bands in the electropherograms (Figure 3). A gradual decrease in the intensity of the bands of self-limited complexes with the increasing concentration of opener O was observed. For the RM-U7/DN-T2 complex, high-intensity bands with mobilities of ~40 bp were detected, as was a lower-intensity band of 240 bp. After the introduction of O, one new band with a mobility of ~100 bp appeared, which corresponded to trimolecular complex V (Figure 3). At 2- and 10-fold excess of O, high-mobility bands with mobilities of ~30–40 bp were detectable, matching oligonucleotide O and self-complementary duplex O_2_ (Figure 3 and Figure S7). The absence of the second additional band representing complex IV was explained by the low quantity of the tetramolecular structures.

In the case of the RM-U3/DN-T2 complex, a single band was observed, presumably being a tetramolecular complex with a mobility of 180 bp (Figure 3, lane 1). The addition of a half amount of the opener to the complex (the ratio of concentrations in the mixture after the addition of the opener, [RM-U3]:[DN-T2]:[O] = 1:1:0.5) diminished the intensity of the original complex and generated two additional bands with mobilities of 210 and 110 bp on the electropherogram (Figure 3, lane 3). These bands corresponded to linear complexes VI and V, respectively. The addition of an excess of the RNA opener produced a complex with a mobility of 100 bp, which matched linear complex V (RM-U3/DN-T2/O) of three oligonucleotides. This finding confirmed the formation of tetramolecular complex III by RM-U3 and DN-T2.

**Figure 4:**
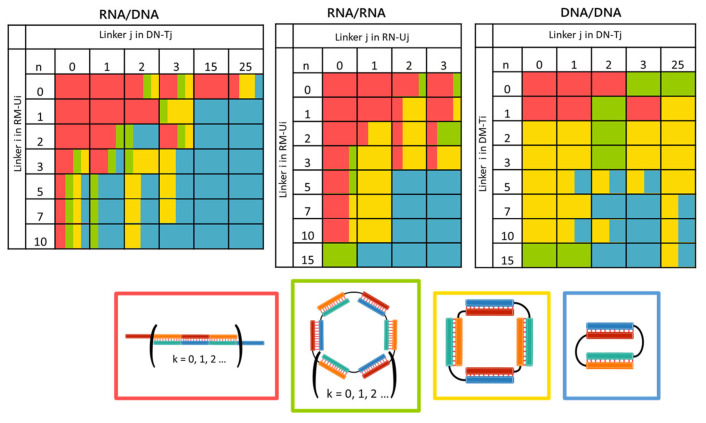
Heat maps illustrating the types of RNA/DNA, RNA/RNA, or DNA/DNA complexes formed by a pair of oligonucleotides with different linker lengths. The colors in the table match the colors of the frames of the complexes below the heat maps: a concatemer (red), a high-molecular-weight self-limited complex (green), a tetramolecular complex (yellow), and a bimolecular complex (blue). In the cells of the table, colors indicate the presence of an expected type(s) of complex on electropherograms. The area of a color in a cell corresponds to the proportion of the respective complex in the NA sample; k is the number of duplexes in a complex; i and j are linker lengths.

Thus, we verified on electropherograms whether bands with mobilities below 170 bp or in the range of 170–260 bp represent the bimolecular and tetramolecular complexes, respectively. This result enabled us to determine the molecularity of hybrid RNA/DNA complexes on the basis of their mobility in the gel shift assay. The mobility and molecularity of the complexes are presented in Tables S4–S6 and summarized as a heat map in Figure 4.

Concatemers were predominantly formed by oligonucleotides without a linker (RM-Ui/DN, i = 0–10, and RM/DN-Tj, j = 0–25). The formation of bimolecular self-limited structures was registered for RM-Ui/DN-Tj complexes (i = 1–10 and j = 15 and 25). Preferential formation of bimolecular complexes was also noted in the case of a linker of 1–3 nt in the DNA strand and a linker of five or more nucleotides in RNA. Tetramolecular complexes predominantly came into being in mixtures with other types of complexes, as did complexes of higher molecular weights. The assembly of high-molecular-weight complexes of various sizes is possible if i and j are greater than 2 in DNA and RNA strands, respectively.

We compared our data with the previously published results on DNA/DNA and RNA/RNA complexes having similar nucleotide sequences. The DNA complexes formed by strands with a linker of 0–1 nt and DM-Ti/DN-Tj (where i = 1, j = 1, and i = 1, j = 3) were concatemers. RM-Ui/RN-Uj complexes with i = 0 and j = 0–3 or j = 0 and i = 0–10 mostly tended to be concatemers in a similar manner.

The RNA complexes with i = 1–3 and j = 1–3 can also form concatemers, typically in a mixture with self-limited complexes of various molecularity levels. The DM-Ti/DN-Tj mixture in many other cases gives rise to tetramolecular complexes, in contrast to the RNA/RNA and RNA/DNA mixtures, which result in bimolecular complexes.

Summarizing the above, the tested oligodeoxyribonucleotides and oligoribonucleotides form different types of complexes. Concatemer structures arise in the case of short linkers in both strands or the absence of a linker in one of the strands. Increasing the linker length led to the folding of the structures into self-limited complexes of different molecularity levels. Shorter linkers yielded mixtures of complexes of various molecularity levels. Longer linkers gave bimolecular complexes. In many ways, the dependence of complex types on linker lengths in the RNA/DNA complex was consistent with a similar dependence for DNA/DNA and RNA/RNA complexes. This similarity between the hybrid and RNA complexes was more pronounced.

### 2.4. Circular Dichroism (CD) Analysis

**Figure 5:**
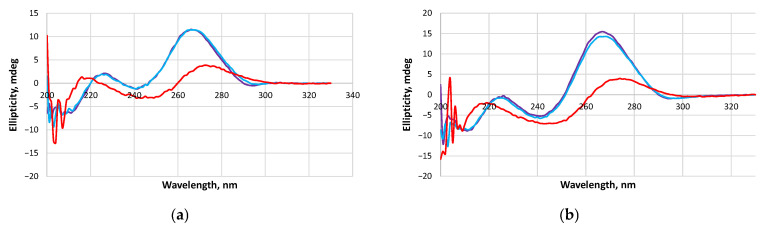
CD spectra of RNA/DNA complexes: (**a**) RM-U7-FAM/DN-T2 (dimer); (**b**) RM-U1-FAM/DN (concatemer) at 5 °C (purple), 25 °C (blue), and 75 °C (red).

Self-assembling NA complexes have the potential to serve as TNAs. One type of biological effect of a TNA is the activation of RNase H [3]. The possibility of RNA degradation in a self-limited hybrid complex or a concatemer hybrid complex by RNase H opens up new opportunities for the design of TNAs. RNA in these complexes can be digested if an RNA/DNA heteroduplex is in a conformation suitable for interaction with RNase H. To characterize the secondary structure, CD spectra were recorded for complexes of different molecularity levels: dimer RM-U7-FAM/DN-T2 and concatemer RM-U1-FAM/DN at different temperatures (5, 25, or 75 °C; Figure 5). When 5 and 25 °C CD spectra are compared, they are the same for each of the two complexes. They have a positive band of high intensity with a maximum of 265 nm and a negative band of much lower intensity with a minimum of 242 nm. The shapes of the spectra are typical for the A-form of RNA/RNA or RNA/DNA duplexes [33].

The similarity of the CD spectra at 5 and 25 °C indicated the complete formation of the complexes at room temperature. The oligonucleotides in the mixture were in a single-stranded state at 75 °C, as confirmed by reduced intensity and altered shapes of the CD spectra. The complex formation and denaturation were confirmed by UV–melting data (Figure S2), melting temperatures (Table 1), and gel shift assays (Figures S4 and S5).

The CD data suggest that the temperature chosen for the gel shift assay was correct. On the other hand, the A-conformation of various RNA/DNA complexes is a prerequisite for the successful digestion of a complex by RNase H.

### 2.5. RNA Digestion Analysis

To evaluate the prospects of the hybrid RNA/DNA concatemer and of self-limited complexes as TNAs, we tested the digestion of RNA in these complexes either by RNase H or by a low-molecular-weight nuclease: imidazole.

Self-limited complexes can be used as carriers of TNAs. In this case, when hybrid RNA/DNA complexes are formed in the cell, RNase H can be activated and digest RNA in such complexes. In the self-limited complexes, duplex segments are spatially close, which can interfere with the functioning of RNase H owing to steric hindrances. We tested the possibility of RNA degradation in hybrid complexes under the action of RNase H to assess the probable usefulness of such complexes in therapeutics.

**Figure 6:**
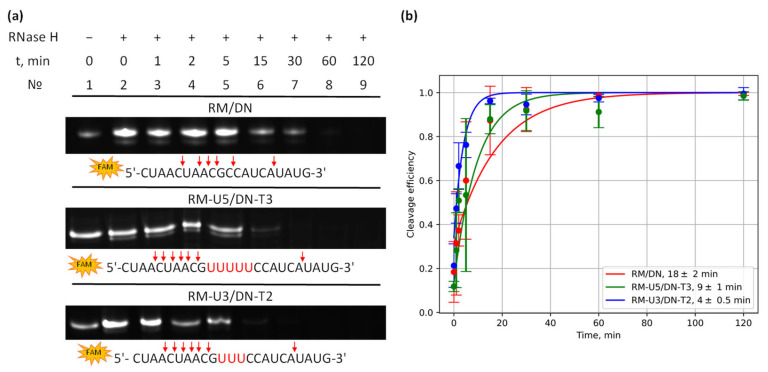
(**a**) Electrophoretic analysis of RNA digestion in complexes RN/DM (concatemer), RM-U5/DN-T3 (dimer), and RM-U3/DN-T2 (tetramer) by RNase H. The red arrow points to digestion sites in the RNA. (**b**) Kinetics of cleavage efficiency (proportion of digested RNA) for different complexes. The time of degradation was determined by fitting the quantitative data on digestion products to an exponential function and is shown in the inset.

RNA degradation in multicomponent complexes by RNase H was analyzed by means of concatemer RM/DN, bimolecular complex RU-U5/DN-T3, and tetramolecular complex RM-U3/DN-T2. Fluorescent label FAM was introduced at the 5′ end of RNA strands to analyze oligoribonucleotide digestion efficiency and their cleavage products. The time dependence of the degradation was examined by denaturing gel electrophoresis (Figure 6 and Figures S8–S10). The assay indicated successful cleavage of an oligoribonucleotide only in duplex segments of the complexes (Figure 6 and Figures S8–S10) in accordance with the specificity of RNase H activity [34]. An uneven distribution of degradation products (NA fragments) was observed across the entire length of the RNA involved in the formation of the double helix with DNA. The RNA cleavage sites were similar among all three types of complexes: cleavage products with a length of 5 to 9 nt and a length of 19 nt predominated. With increasing reaction time for RM-U5-FAM/DN-T3, additional cleavage products corresponding to oligomers 17, 19, and 20 nt long emerged. This phenomenon was caused by further degradation of the hydrolysis products.

A kinetic analysis suggested that the digestion of FAM-labeled RNA in a concatemer (RM-FAM/DN), bimolecular (RM-U5-FAM/DN-T3), and tetramolecular (RM-U3-FAM/DN-T2) complexes proceeded almost completely within 30 min. The time dependence of the amount of intact RNA allowed us to determine the time of degradation via fitting of kinetic curves to an exponential function. A degradation time of 10 ± 7 min was found for the studied complexes (Figure 6b). Standard deviation at each data point in Figure 6b was obtained by quantitative analysis of the bands in the electropherograms via averaging of at least two replicated experiments (Figures S8–S10).

The main contribution to the magnitude of systematic error is made by the reproducibility of an experiment (Figures S8–S10). Effective digestion of RNA in the RNA/DNA concatemer and self-limiting complexes indicates the ability to recruit RNase H when such complexes are used as TNAs.

aRNases contain two components: oligonucleotides as motifs that recognize specific RNA sequences and catalytic group (s) for RNA degradation. The classic mechanism of RNA degradation includes a rate-limiting step: acquisition of the “in-line” conformation by an RNA cleavage site, e.g., [34,35]. We propose that the formation of the self-limited complexes may increase the probability of the “in-line” conformation within the linker segments. We decided to use bi- and tetramolecular complexes to test this hypothesis.

**Figure 7:**
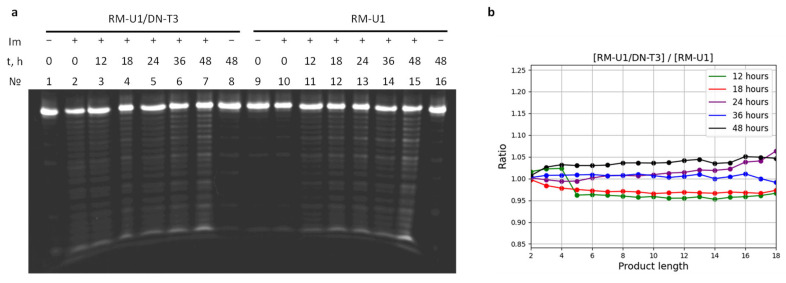
(**a**) Electrophoretic analysis of RNA digestion in the RM-U5/DN-T3 complex (dimer, lanes 2–7) and single-stranded state RM-U5 (lanes 10–15) by imidazole. Lanes 1 (0 h) and 8 (48 h) represent a complex in water without the addition of imidazole; lanes 2–8 and 10–15 correspond to different digestion durations: 2: 0 h, 3: 12 h, 4: 18 h, 5: 24 h, 6: 36 h, 7: 48 h, 10: 0 h, 11: 12 h, 12: 18 h, 13: 24 h, 14: 36 h, and 15: 48 h. SsRNA stability in water without the addition of imidazole: lane 9: 0 h, lane 16: 48 h. (**b**) The ratio of the areas under every peak for an RNA in the complex and the RNA in a single-stranded state.

One of the popular types of catalytic components of aRNases is imidazole derivatives [36]. The mechanism of action of imidazole toward RNA is well studied [37]. We investigated RNA degradation in dimer complexes (RM-U5-FAM/DN-T3 and RM-U7-FAM/DN-T2) and tetramer complexes (RM-U1-FAM/DN-T3 and RM-U3-FAM/DN-T2) by imidazole. The single-stranded RNA (ssRNA) corresponding to each complex served as a reference. RNAs in both the single-stranded state and in the complex were digested by means of a 2 M solution of imidazole, and the products were analyzed by gel electrophoresis (Figure 7 and Figures S11–S14). Quantitative analysis revealed nonuniform degradation along the strands. Calculated ratios of quantities of the RNA digestion products (RNA fragments) in each band of a complex to such values of ssRNA at specific time points are shown in Figure 7 and Figures S11–S14. One can see that there are no significant differences in band intensity between self-limited complexes and control ssRNAs. Examination of the RNA degradation kinetics by fitting the quantitative data on digestion products to an exponential function yielded similar degradation times. These findings implied that the RNA degradation in the complexes proceeded without preferential degradation in the ssRNA regions of the complexes.

Thus, the analysis of the RNA digestion by RNase H and imidazole in the self-limited and concatemer complexes pointed to the probable usefulness of these complexes as TNAs.

## 3. Materials and Methods

### 3.1. Materials

All solvents and other reagents were purchased from Sigma (St. Louis, MO, USA) at the highest available grade and used without purification.

The oligonucleotides were obtained from the High-accuracy RNA/DNA Synthesis Core Facility at the Institute of Chemical Biology and Fundamental Medicine, the Siberian Branch of the Russian Academy of Sciences (ICBFM SB RAS, Novosibirsk, Russia). They were synthesized by the phosphoramidite method from commercially available RNA/DNA (Glen Research, Sterling, VA, USA) and fluorescein (FAM) phosphoramidite 6-isomer (Lumiprobe, Moscow, Russia) synthons on an ASM-800 synthesizer (Biosset, Novosibirsk, Russia) and purified by gel electrophoresis.

### 3.2. Oligonucleotide Concentration Determination

UV spectroscopy on a Cary 3500 spectrophotometer (Agilent, Santa Clara, CA, USA) was applied for the oligonucleotides’ quantification. We calculated the extinction coefficient at 260 nm as described in Ref. [38].

### 3.3. UV Melting Analysis

This analysis was performed on the Cary 3500 spectrophotometer (Agilent, Santa Clara, CA, USA). Wavelengths of 260, 270, and 330 nm were employed for this purpose [39]. A buffer consisting of 100 mM NaCl, 10 mM C_2_H_6_AsO_2_Na (sodium cacodylate), and 15 mM MgCl_2_, pH 7.2 was used for UV melting. For thermodynamic stabilization of duplexes, we chose magnesium ions at low concentrations rather than monovalent cations at high concentrations to achieve the same stabilizing effect. The reason for the choice of the divalent ion is that at high ion concentrations, there would be problems in the gel shift assay and RNA enzymatic digestion. The concentration of every oligomer in the solution was 1 μM. Heating and cooling experiments were carried out in the temperature range of 5–95 °C at a rate of 0.5 °C/min in 0.2 cm quartz cells.

### 3.4. CD Spectroscopy

This method was used to characterize RNA/DNA complexes’ secondary structure. A J-600 spectropolarimeter (Jasco, Tokyo, Japan) was utilized for this purpose. The experiment was conducted in the range of 200–330 nm at a resolution of 1 nm, a bandwidth of 2 nm, a sensitivity of 50 millidegrees, a scanning speed of 50 nm/min, and five replications, and their data were averaged. A thermostatted quartz cell with an optical path length of 1 cm was used. The same buffer was chosen as in the UV melting experiments: 100 mM NaCl, 10 mM sodium cacodylate, and 15 mM MgCl_2_. CD spectra were acquired at 5, 25, and 75 °C. The cell was thermostatted by a CC3 water bath thermostat (Huber, Bergheim, Germany).

### 3.5. The Gel Shift Assay

To determine the complexes’ mobility in a gel and of their molecularity, the gel shift assay was performed in a 15% polyacrylamide gel (PAAG; acrylamide and N,N′-methylene bisacrylamide in a ratio of 39:1) in an electric field of 15 V/cm in Tris-borate (TB) buffer [89 mM tris (hydroxymethyl)aminomethane, 89 mM boric acid, pH 8.3] supplemented with 15 mM MgCl_2_. Gels were thermostatted at 5 °C by the CC3 water bath thermostat (Huber, Germany). Xylene cyanol was utilized for visual tracking of complexes’ migration in a gel. Stains-all (Sigma, USA) was used to stain each gel. A double-stranded DNA ladder of 50–1500 bp (SibEnzyme, Novosibirsk, Russia) was employed for the analysis of complexes′ mobility. Samples (10 μL) were prepared in a buffer composed of 10 mM sodium cacodylate, 100 mM NaCl, and 15 mM MgCl_2_. The concentration of RM or DN series oligonucleotides was 10 μM.

### 3.6. RNA Digestion

Reaction mixtures containing DNA and FAM-labeled RNA oligonucleotides at a concentration of 1 µM were prepared for the analysis of RNA digestion by either RNase H or imidazole. The assay of RNA degradation by RNase H (Transgene, Shanghai, China) was performed in a buffer consisting of 200 mM Tris-HCl, 150 mM dithiothreitol (DTT), 1 M KCl, and 45 mM MgCl_2_. Cleavage efficiency was assessed in 2 M imidazole solutions, pH ~7.5, with the addition of 10 mM MgCl_2_ for the efficient formation of complexes.

Aliquots (10 µL) at various time points (either 0, 1, 2, 5, 15, 30, 60, and 120 min at 30 °C for RNase H or 0, 12, 18, 24, 36, and 48 h at 25 °C for the imidazole solution) were taken from the microcentrifuge tubes during incubation of the reaction mixture. Next, the aliquots were placed in 500 µL of 2% LiClO_4_ in acetone for precipitation. After 1 h in the cold (−20 °C), the solutions were centrifuged at 5 °C and 13,000× *g* for 3 min, and the supernatant was removed. Then, the pellets were washed with 200 µL of acetone and air dried. Each precipitate was analyzed by gel electrophoresis.

The electrophoretic analysis was performed under denaturing conditions in a 24% PAAG (acrylamide and N,N′-methylene bisacrylamide in a ratio of 29:1) in a TB buffer [89 mM tris (hydroxymethyl)aminomethane, 89 mM boric acid, 8 M urea, pH 8.3] in an electric field of 15 V/cm. Bromophenol blue was utilized for visual tracking of sample migration. Gels were scanned using a VersaDoc gel documentation system (Bio-Rad, Hercules, CA, USA).

Quantitative analysis of the data obtained by the gel electrophoresis was performed using our custom-designed Python program (Python 3.11.8) with the help of libraries matplotlib, scipy, and numpy to analyze electropherogram pixels’ brightness.

## 4. Conclusions

In this paper, we analyzed the formation of hybrid RNA/DNA concatemers and self-limited complexes by a pair of oligonucleotides. UV melting analysis showed the stabilization of linear concatemers by stacking interactions within nicks. This phenomenon also resulted in asymmetric shapes of UV melting profiles and higher melting temperatures as compared to the self-limited complexes. Similar results have been obtained previously for DNA/DNA and RNA/RNA constructs [24,25]. Effects of the size of the linker between duplex-forming segments on the shape and molecularity of the complexes were compared and systematized for RNA/DNA, DNA/DNA, and RNA/RNA assemblies. We found that the different NAs could form complexes of various types and molecularity levels. Shorter linkers lead to the formation of complexes of mixed molecularity. Longer linkers preferentially give bimolecular complexes. In many ways, the dependence of complex types on the length of the oligonucleotide linker is similar between RNA/DNA complexes and DNA/DNA (and RNA/RNA) complexes. This similarity is more pronounced between the hybrid and RNA/RNA complexes because of the A-form of their duplex segments.

We compared the types of complexes and the melting temperatures (Figure S15). Typically, T_m_ is higher for concatemer complexes. This is caused by a stacking interaction within a nick [28,40]. For other complexes, no strong correlation of the complex type with thermal stability was observed.

We systematized the data on the complexes’ types as a heat map, making the obtained data a convenient tool for the design of an NA construct of desired geometry. General rules for the formation of RNA/DNA, DNA/DNA, and RNA/RNA complexes were figured out, and the feasibility of changing their structure and molecularity—via the strand displacement mechanism by the addition of an opener and a closer—was demonstrated. This makes it possible not only to design but also to purposefully alter the structural characteristics of such complexes, depending on the task at hand.

CD analysis pointed to the A-form of the double helix in RNA/DNA complexes of various molecularity levels. This finding makes RNA suitable for digestion by RNase H in these complexes. We showed RNase H-driven efficient and specific digestion of RNA within hybrid–duplex segments of a concatemer complex and self-limited bi- and tetramolecular complexes. Moreover, in hybrid complexes, we noticed efficient degradation of RNA by a concentrated imidazole solution. These data hold promise for the design of novel TNAs [4,5,21,22,23]. For instance, DNA-based TNAs can be delivered into cells by means of multicomponent complexes as carriers. The promise of concatemer NA complexes has been demonstrated previously [41,42]. Self-limited NA complexes could be used for the simultaneous delivery of multiple therapeutic oligonucleotides into the cell in a similar manner.

The cell’s functional RNA is expected to bind to DNA, resulting in the proposed self-limited structures, where subsequent digestion by RNase H may occur. Moreover, a certain RNA sequence can be digested in such complexes if the DNA strand in the complex is covalently attached to a catalytically active center (s) (of aRNase), such as those proposed earlier (e.g., [42,43]). Thus, the hybrid self-limited complexes are good candidates for TNAs.

The current work offers a new convenient approach to the design of supramolecular complexes as tools for nanotechnology, nanomachines, and biomedical applications.

## Figures and Tables

**Table 1:** Melting temperatures (T_m_, °C) ^1^ obtained by UV melting analysis at a 1 μM concentration of oligonucleotides.

	DN	DN-T1	DN-T2	DN-T3	DN-T15	DN-T25
**RM**	47.8	43.5	43.6	46.0	45.6	49.4
**RM-U1**	41.7	38.6	36.8	40.4	42.0	43.4
**RM-U2**	43.7	40.4	39.0	42.1	42.9	43.0
**RM-U3**	40.8	38.8	37.4	41.2	42.6	41.9
**RM-U5**	40.7	38.4	38.2	42.0	42.7	42.5
**RM-U7**	40.6	39.1	39.8	42.0	42.2	42.0
**RM-U10**	41.0	39.0	40.8	43.2	42.6	42.2

^1^ Standard error of T_m_ determination is ±0.5 °C.

## Data Availability

Raw data are available from the corresponding author upon reasonable request.

## Supplementary Materials

The following supporting information can be downloaded at https://www.mdpi.com/article/10.3390/molecules29245896/s1, Figure S1: UV melting curves (at 260 nm) of the RM-U2/DN-T1 complex during heating and cooling; Figure S2: Differential UV melting curves of RM-Ui/DN-Tj complexes (i = 0, 1, 2, 3, 5, 7, or 10; j = 0, 1, 2, 3, 15, or 25); Figure S3: Differential UV melting curves of RM-Ui-FAM/DN-Tj complexes (i = 0, 1, 3, 5, 7, or 10; j = 0, 1, 2, or 3); Figures S4–S6: The gel shift assay of oligonucleotide complexes RM-Ui/DN and RM-Ui/DN-T1 with different lengths of linkers; Figure S7: The gel shift assay of opener O at different concentrations; Figures S8–S10: Electrophoretic analysis of RNA digestion in the RM/DN complex (concatemer) by RNase H; Figures S11–S14: Electrophoretic analysis of RNA digestion in the RM-Ui/DN-Tj complex and results of the electropherograms’ analysis; Table S1: Code names, characteristics, and sequences of the tested oligonucleotides; Table S2: Melting temperatures (T_m_, °C) obtained by the UV melting analysis at a 1 μM concentration of oligonucleotides; Table S3: Differences in melting temperatures (T_m_, °C) of FAM-labeled and native RNA complexes with DNA, as determined by the UV melting analysis at a 1 μM concentration of oligonucleotides; Tables S4–S6: Mobility, complex types, and sizes determined by gel shift assays of oligonucleotide complexes RM-Ui/DN-Tj with different lengths of linkers; Figure S15: A comparison of the complex types and the melting temperatures.
