# Supplementary material for: Hybrid RNA/DNA Concatemers and Self-Limited Complexes: Structure and Prospects for Therapeutic Applications
Source: Molecules. 2024 Dec 13;29(24):5896. doi: 10.3390/molecules29245896 (PMC11677838; doi:10.3390/molecules29245896)
Supplement: Supplementary file 1 [file molecules-29-05896-s001.zip › molecules-3309359-supplementary.pdf]

SUPPORING INFORMATON

Hybrid RNA/DNA Concatemers and Self-Limited Complexes: Structure and Prospects for Therapeutic Applications

Maria A. Kanarskaya [1,2], Sofia V. Novikova [1,2] and Alexander A. Lomzov [1,2,*]

[1] Institute of Chemical Biology and Fundamental Medicine SB RAS, Novosibirsk 630090, Russia
[2] Department of Physics, Novosibirsk State University, Novosibirsk 630090, Russia
* Correspondence: lomzov@niboch.nsc.ru; Tel.: +7-913-009-2889

Table of Contents

Model System 3

Thermal Denaturation Analysis 4

Gel Shift Assay Analysis 7

RNA Digestion Analysis 11

Model System

Table S1. Codes, characteristics and sequences of the tested oligonucleotides. The red letters in the M and N sequences denote the linker; in the opener, they denote the overhangs.

#	Code	Sequence, 5′ → 3′
1	RM	r[CUAACUAACGCCAUCAUAUG]
2	RM-U1	r[CUAACUAACG**U**CCAUCAUAUG]
3	RM-U2	r[CUAACUAACG**UU**CCAUCAUAUG]
4	RM-U3	r[CUAACUAACG**UUU**CCAUCAUAUG]
5	RM-U5	r[CUAACUAACG**UUUUU**CCAUCAUAUG]
6	RM-U7	r[CUAACUAACG**UUUUUUU**CCAUCAUAUG]
7	RM-U10	r[CUAACUAACG**UUUUUUUUUU**CCAUCAUAUG]
8	RM-FAM	FAM - r[CUAACUAACGCCAUCAUAUG]
9	RM-U1-FAM	FAM - r[CUAACUAACG**U**CCAUCAUAUG]
10	RM-U3-FAM	FAM -r[CUAACUAACG**UUU**CCAUCAUAUG]
11	RM-U5-FAM	FAM -r[CUAACUAACG**UUUUU**CCAUCAUAUG]
12	RM-U7-FAM	FAM -r[CUAACUAACG**UUUUUUU**CCAUCAUAUG]
13	RM-U10-FAM	FAM -r[CUAACUAACG**UUUUUUUUUU**CCAUCAUAUG]
14	DN	CGTTAGTTAGCATATGATGG
15	DN-T1	CGTTAGTTAG**T**CATATGATGG
16	DN-T2	CGTTAGTTAG**TT**CATATGATGG
17	DN-T3	CGTTAGTTAG**TTT**CATATGATGG
18	DN-T15	CGTTAGTTAG**TTTTTTTTTTTTTTT**CATATGATGG
19	DN-T25	CGTTAGTTAG**TTTTTTTTTTTTTTTTTTTTTTTTT**CATATGATGG
20	O	r[**AAAAA**CGUUAGUUAG]

Thermal Denaturation Analysis

Figures S1-S3 show the results of UV melting analysis of RNA/DNA complexes. Figure S1 shows the UV melting curve, and Figures S2-3S show the differential UV melting curves calculated as the first derivative of optical absorbance with respect to temperature. The x-axis in Figures S1-S3 is temperature, the y-axis in Figure S1 is optical density at 260 nm wavelength (A260), and in Figures S2-S3 is the ratio of optical density to temperature (ΔABS/ΔT). o.u. - Optical units. The buffer conditions: 100 mM NaCl, 10 mM C2H6AsO2Na, 15 mM MgCl2, pH 7.2. The concentration of every oligomer in the solution was 1 μM.

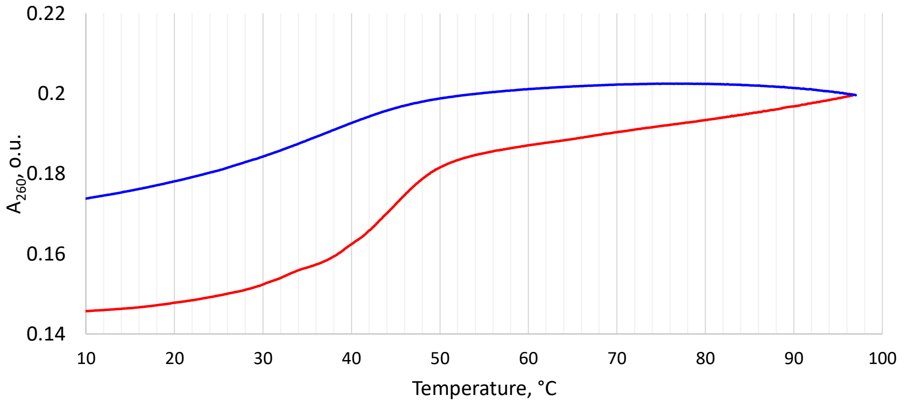

Figure S1. UV melting curves at 260 nm (optical absorption at 260 nm A_{260} in temperature) of RM-U2/DN-T1 complex during heating (red) and cooling (blue).

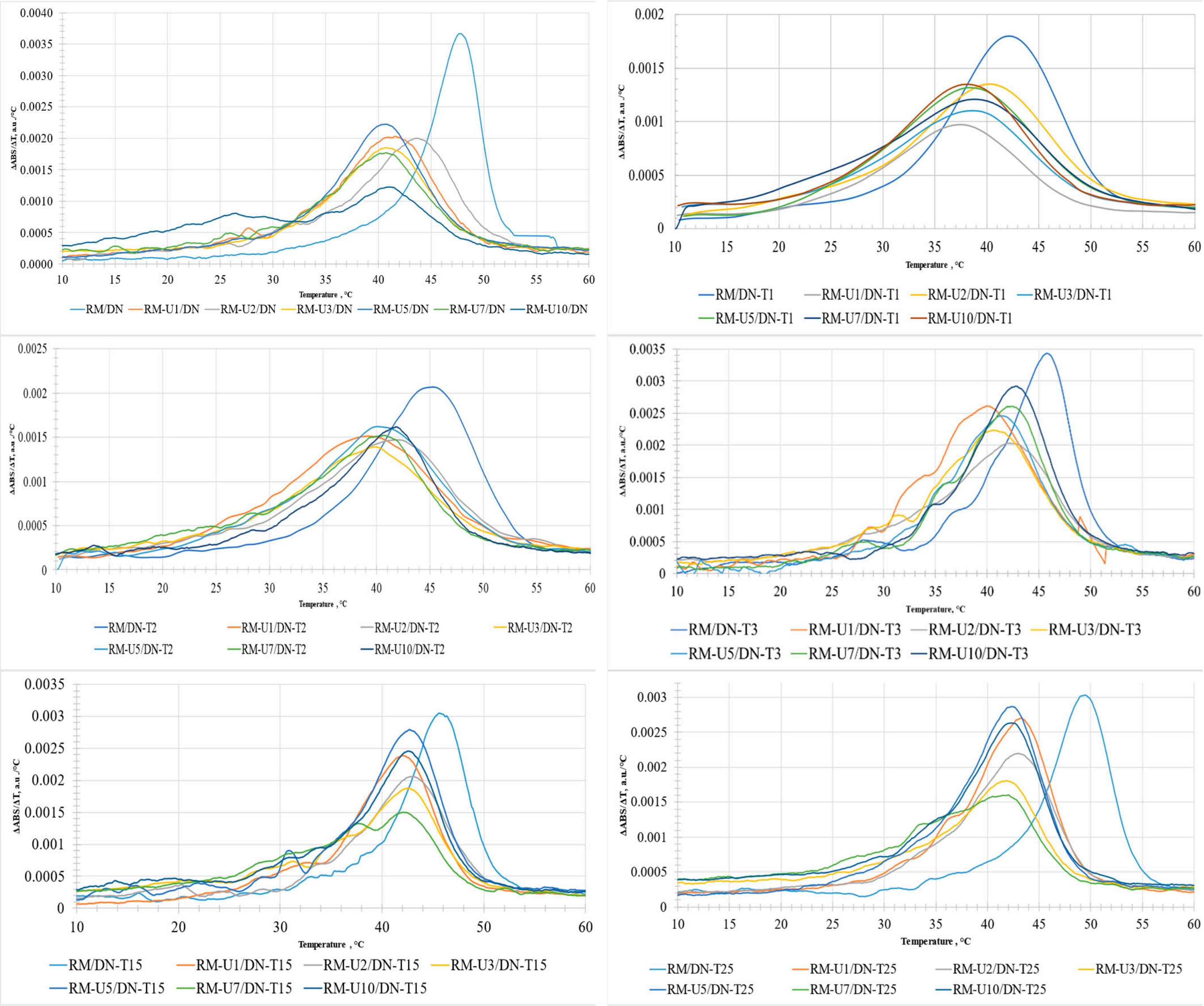

Figure S2. Differential UV melting curves (first derivative of optical absorption on temperature ΔABS/ΔT) of RM-Ui/DN-Tj complexes (i = 0, 1, 2, 3, 5, 7, 10; j = 0, 1, 2, 3, 15, 25)

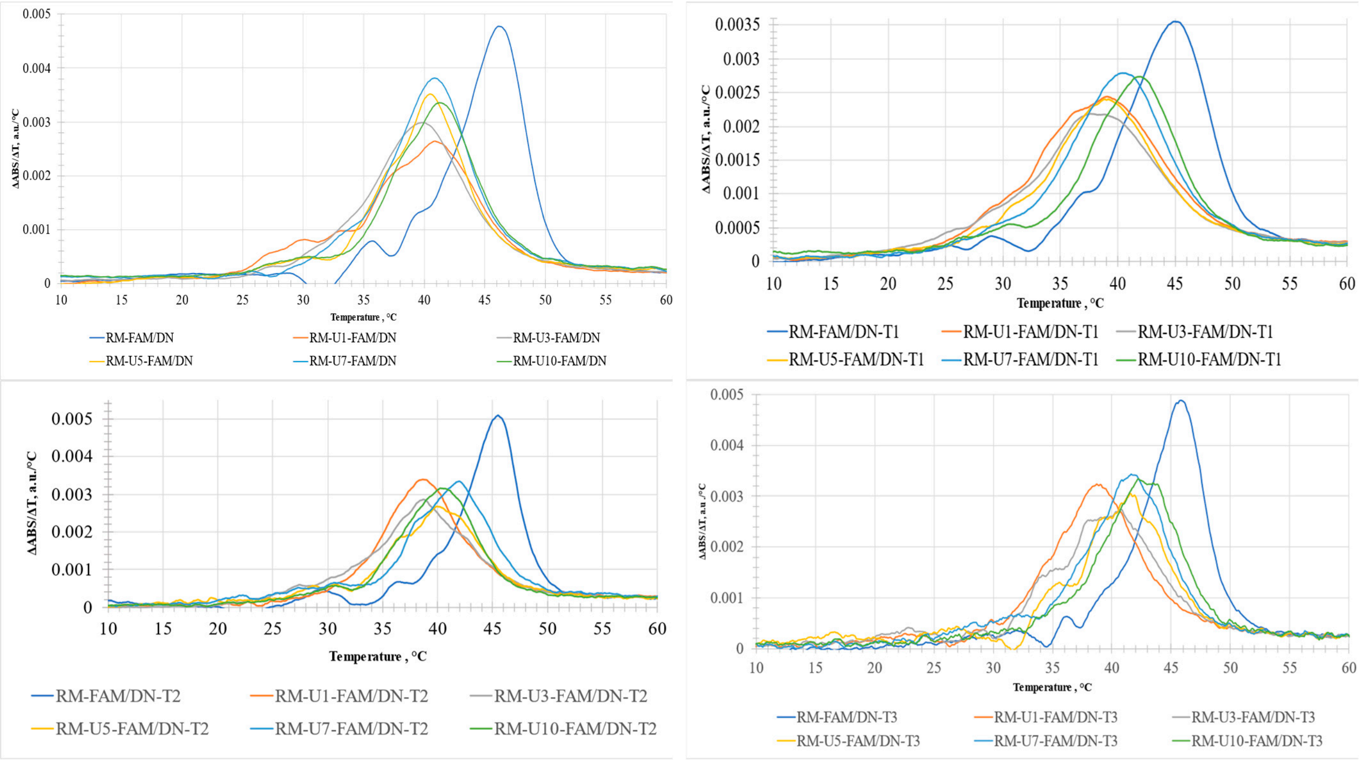

Figure S3. Differential UV melting curves (first derivative of optical absorption ΔABS/ΔT on temperature) of RM-Ui-FAM/DN-Tj complexes (i = 0, 1, 3, 5, 7, 10; j = 0, 1, 2, 3).

Table S2. Melting temperature values (Tm, °C) obtained by UV-melting analysis at 1 μM oligonucleotides' concentration

	DN	DN-U1	DN-U2	DN-U3
**RM-FAM**	46.3	45.2	45.8	45.9
**RM-U1-FAM**	40.8	38.7	39.0	38.3
**RM-U3-FAM**	40.0	38.5	38.8	39.5
**RM-U5-FAM**	40.8	39.5	40.6	41.0
**RM-U7-FAM**	41.0	40.8	42.1	41.7
**RM-U10-FAM**	41.3	42.0	40.8	43.0

Table S3. Differences in melting temperature values (Tm, °C) of FAM-labeled and native RNA complexes with DNA obtained by UV-melting analysis at 1 μM oligonucleotides' concentration

	DN	DN-U1	DN-U2	DN-U3
**RM-FAM**	-1.5	1.7	2.2	0.9
**RM-U1-FAM**	-0.9	0.1	2.2	-0.5
**RM-U3-FAM**	-0.8	-0.3	1.4	-0.3
**RM-U5-FAM**	0.1	1.1	2.4	0.2
**RM-U7-FAM**	0.4	1.7	2.3	-0.2
**RM-U10-FAM**	0.3	3.0	0.0	1.3

Gel Shift Assay Analysis

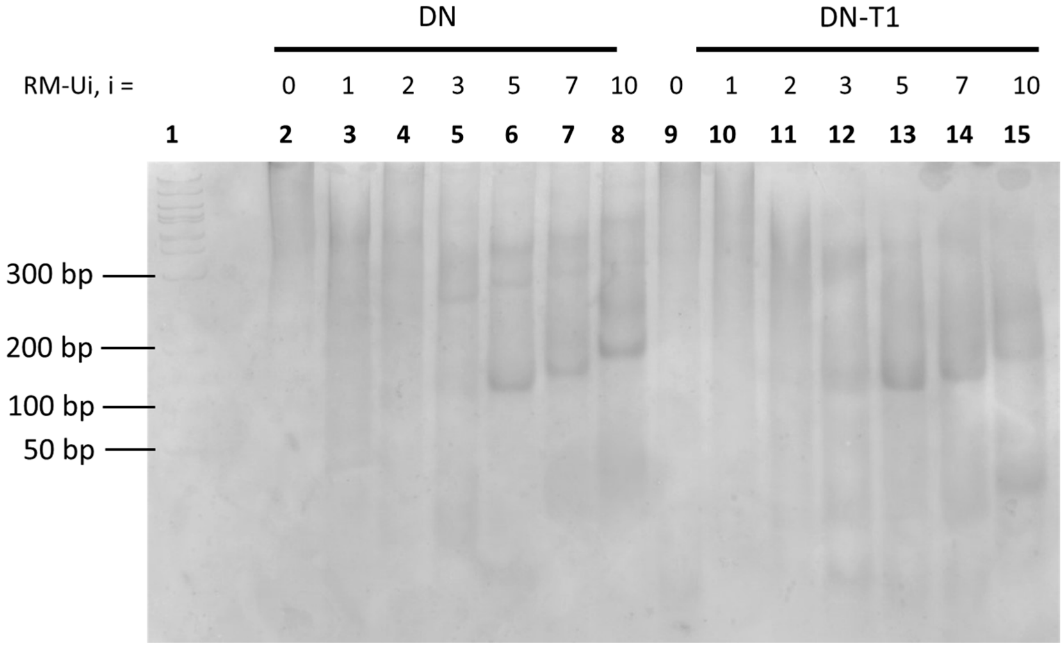

Figure S4. The gel shift assay of oligonucleotides' complexes RM-Ui/DN and RM-Ui/DN-T1 with different lengths of linkers (i = 0, 1, 2, 3, 5, 7, 10). A dsDNA ladder of 50–1000 bp is shown on the left. The buffer conditions: 100 mM NaCl, 10 mM $C_2H_6AsO_2Na$, 15 mM $MgCl_2$, the concentration of every oligomer in the solution was 1 µM.

Table S4. Mobility, complex type, and size determined by analysis gel shift assays for oligonucleotides' complexes RM-Ui/DN and RM-Ui/DN-T1 with different lengths of linkers (i = 0, 1, 2, 3, 5, 7, 10). Designations**:** "conc" - concatameric complexes, "2" - bimolecular complexes, "4" - tetramolecular complexes, "6" - complexes of molecularity higher than 4., "c" - a distributed signal in the lane. The number in parentheses indicates the less pronounced band in the lane, i.e. a smaller amount of the desired type of complex in the mixture.

№	Sample	Mobility, bp	Types of complexes
1	dsDNA ladder	-	-
2	RM/DN	c	conc
3	RM-U1/DN	c	conc
4	RM-U2/DN	c	conc
5	RM-U3/DN	250, (400), c	4, (6), conc
6	RM-U5/DN	120, (280), (400), c	2, (4), (6), conc
7	RM-U7/DN	150, (300), (500), c	2, (4), (6), conc
8	RM-U10/DN	190, (300), (600), c	2, (4), (6), conc
9	RM/DN-T1	c	conc
10	RM-U1/DN-T1	c	conc
11	RM-U2/DN-T1	(600), c	(6), conc
12	RM-U3/DN-T1	120, (380), c	2, (6), conc
13	RM-U5/DN-T1	120, (400)	2, (6)
14	RM-U7/DN-T1	150, (440)	2, (6)
15	RM-U10/DN-T1	190, (440)	2, (6)

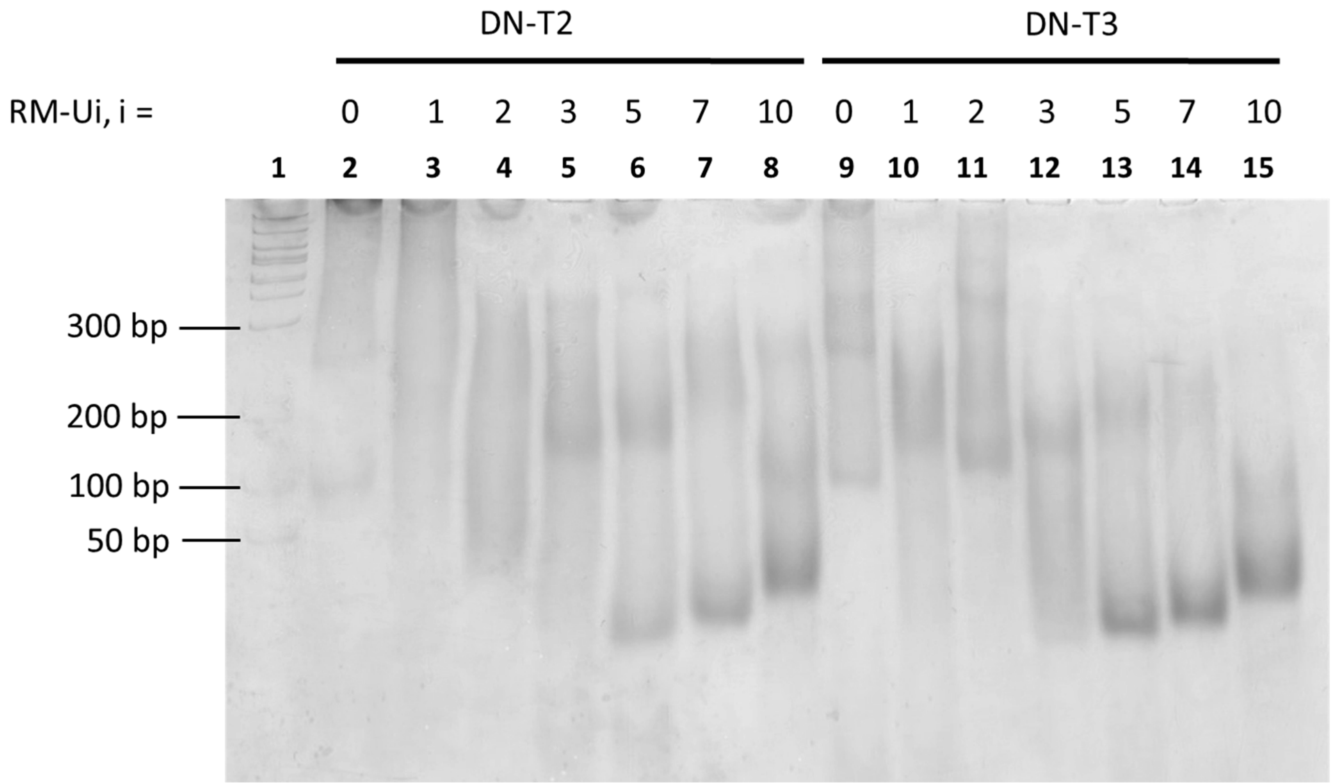

Figure S5. The gel shift assay of oligonucleotides' complexes RM-Ui/DN-T2 and RM-Ui/DN-T3 with different lengths of linkers (i = 0, 1, 2, 3, 5, 7, 10). A dsDNA ladder of 50–1000 bp is shown on the left. The buffer conditions: 100 mM NaCl, 10 mM $C_2H_6AsO_2Na$, 15 mM $MgCl_2$, the concentration of every oligomer in the solution was 1 μM.

Table S5. Mobility, complex type, and size determined by analysis gel shift assays for oligonucleotides' complexes RM-Ui/DN-T2 and RM-Ui/DN-T3 with different lengths of linkers (i = 0, 1, 2, 3, 5, 7, 10). Designations: "conc" - concatameric complexes, "2" - bimolecular complexes, "4" - tetramolecular complexes, "6" - complexes of molecularity higher than 4., "c" - a distributed signal in the lane. The number in parentheses indicates the less pronounced band in the lane, i.e. a smaller amount of the desired type of complex in the mixture.

№	Sample	Mobility, bp	Types of complexes
1	dsDNA ladder	-	-
2	RM/DN-T2	(100), (260), c	(4), (6), conc
3	RM-U1/DN-T2	c	conc
4	RM-U2/DN-T2	50, 350	2, 6
5	RM-U3/DN-T2	180, (350)	4, (6)
6	RM-U5/DN-T2	35, 200	2, 4
7	RM-U7/DN-T2	40, (240)	2, (4)
8	RM-U10/DN-T2	45, (280)	2, (4)
9	RM/DN-T3	(100), (260), (400), c	(4), (6), (8), conc
10	RM-U1/DN-T3	180, (260)	4, (6)
11	RM-U2/DN-T3	(100), (280), (400), c	(4), (6), (8), conc
12	RM-U3/DN-T3	(35), 180	(2), 4
13	RM-U5/DN-T3	35, (200)	2, (4)
14	RM-U7/DN-T3	40, (200)	2, (4)
15	RM-U10/DN-T3	45	2

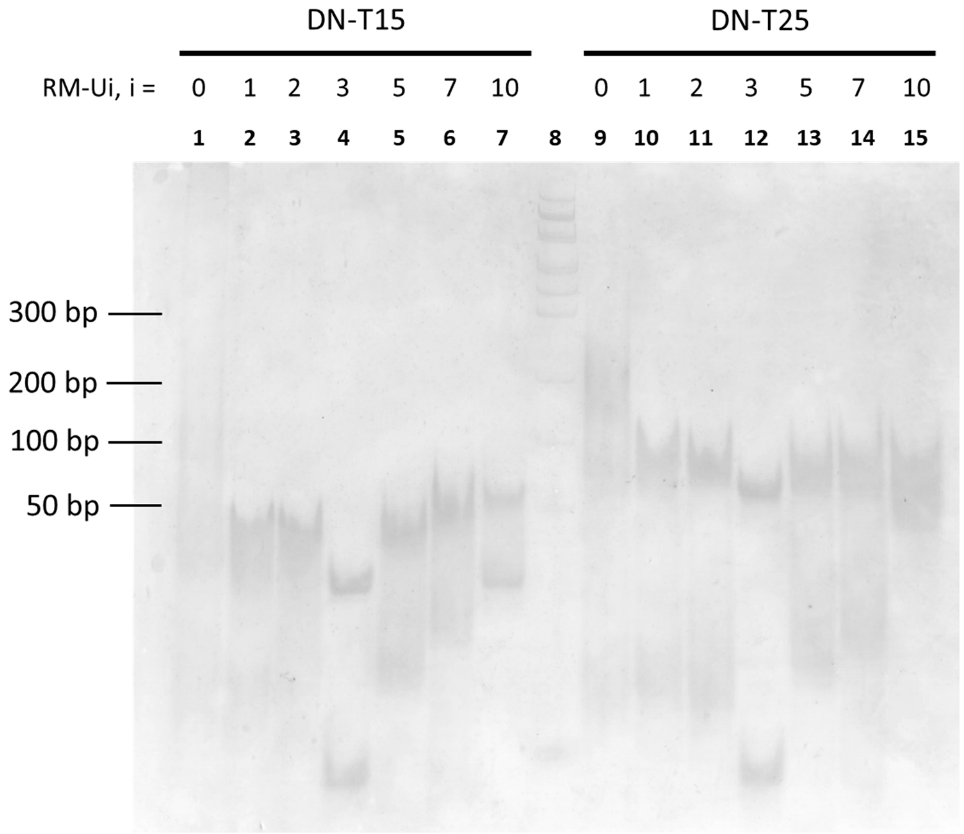

Figure S6. The gel shift assay of oligonucleotides' complexes RM-Ui/DN-T15 and RM-Ui/DN-T25 with different lengths of linkers (i = 0, 1, 2, 3, 5, 7, 10). A dsDNA ladder of 50–1000 bp is shown in the middle. The buffer conditions: 100 mM NaCl, 10 mM $C_2H_6AsO_2Na$, 15 mM $MgCl_2$, the concentration of every oligomer in the solution was 1 μM.

Table S6. Mobility, complex type, and size determined by analysis gel shift assays for oligonucleotides' complexes RM-Ui/DN-T15 and RM-Ui/DN-T25 with different lengths of linkers (i = 0, 1, 2, 3, 5, 7, 10). Designations**:** "conc" - concatameric complexes, "2" - bimolecular complexes, "4" - tetramolecular complexes, "6" - complexes of molecularity higher than 4., "c" - a distributed signal in the lane. The number in parentheses indicates the less pronounced band in the lane, i.e. a smaller amount of the desired type of complex in the mixture.

№	Sample	Mobility, bp	Types of complexes
1	RM/DN-T15	c	conc
2	RM-U1/DN-T15	45	2
3	RM-U2/DN-T15	45	2
4	RM-U3/DN-T15	40	2
5	RM-U5/DN-T15	45	2
6	RM-U7/DN-T15	50	2
7	RM-U10/DN-T15	60	2
8	dsDNA ladder	-	-
9	RM/DN-T25	(90), 200, c	(2), 4, conc
10	RM-U1/DN-T25	90	2
11	RM-U2/DN-T25	90	2
12	RM-U3/DN-T25	60	2
13	RM-U5/DN-T25	90	2
14	RM-U7/DN-T25	90	2
15	RM-U10/DN-T25	90	2

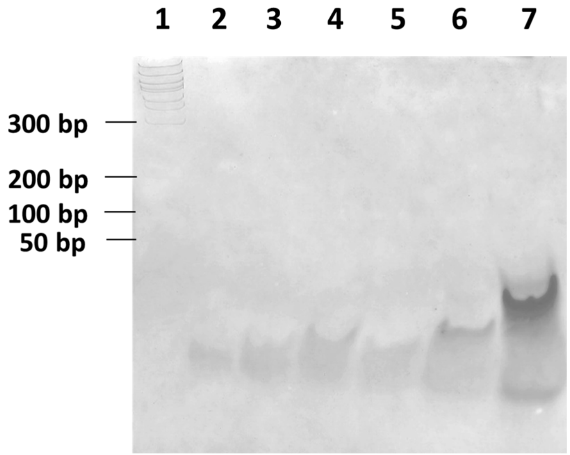

Figure S7. The gel shift assay of opener O at different concentrations. Lines: 1—dsDNA ladder; 2—2.5 µM; 3—5 µM; 4—7.5 µM; 5—10 µM; 6—20 µM; 7—100 µM. A dsDNA ladder of 50–1000 bp is shown on the left. The buffer conditions: 100 mM NaCl, 10 mM $C_2H_6AsO_2Na$, 15 mM $MgCl_2$.

RNA Digestion Analysis

This section presents data of electophoretic analysis of RNA degradation by RNase H (Figures S8-S10) and by imidazole (Figures S11-S14). The buffer condition for the RNase H digestion analysis was 200 mM Tris-HCl, 150 mM DTT, 1 M KCl, 45 mM $MgCl_2$. The buffer conditions for the imidazole digestion analysis was 2M imidazole solutions, 10 mM $MgCl_2$, pH ~7.5. The concentration of every oligomer in the solution was 1 μM.

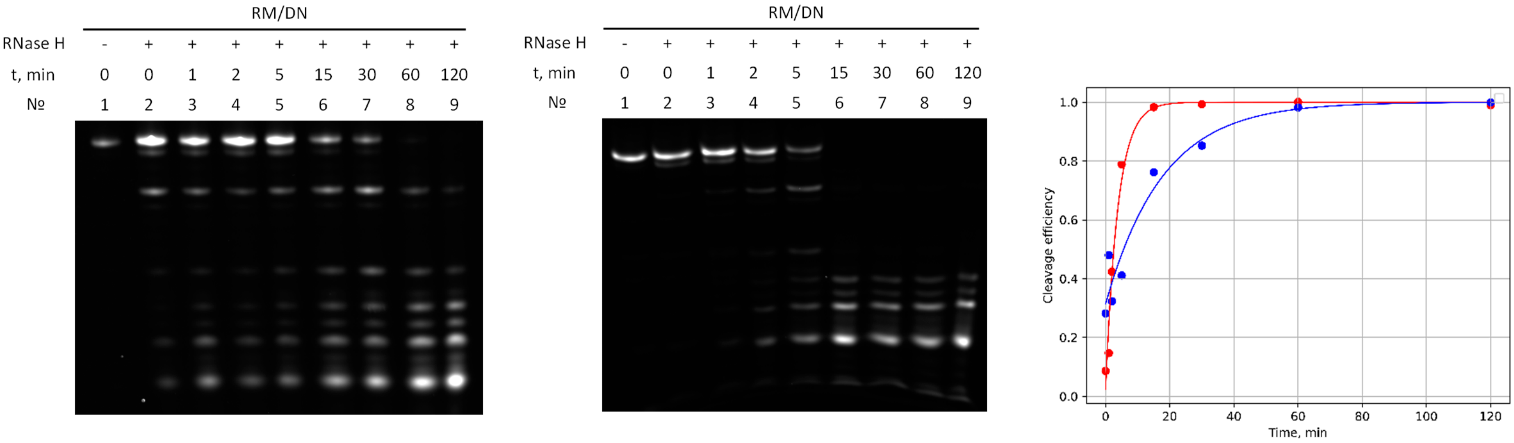

Figure S8. Electrophoretic analysis of RNA digestion in the complex RM/DN (concatamer) by RNase H. The left and middle figures are a repetition of the experiment. Line 1 is a complex in water without the addition of RNase H, lines 2-9 are a complex interacting with RNase H at different time intervals. (Right) Kinetics of cleavage efficiency (proportion of digested RNA) for different experiments. The red curve corresponds to the left figure, the blue curve to the middle figure.

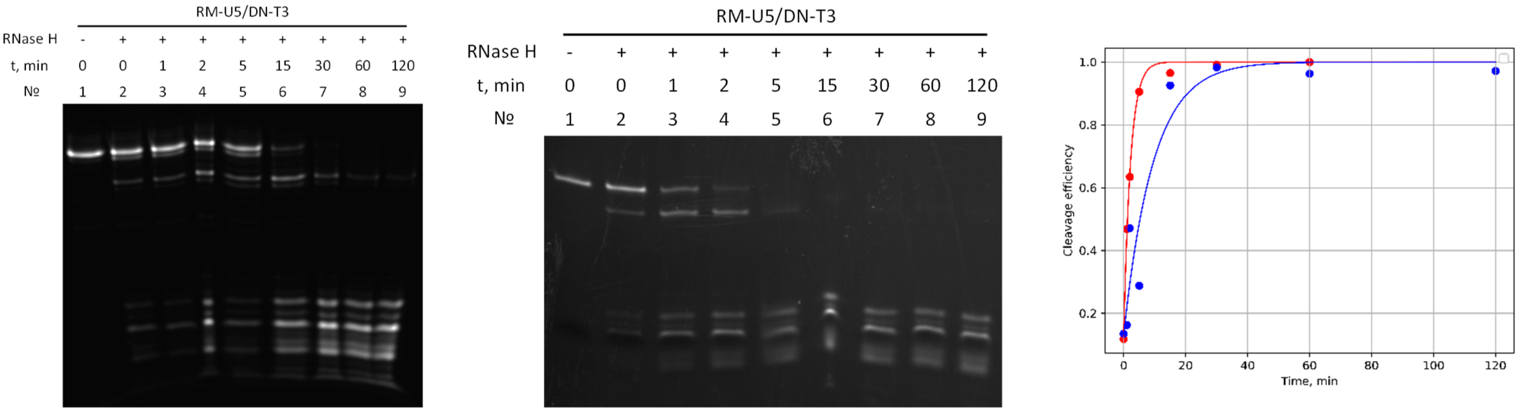

Figure S9. Electrophoretic analysis of RNA digestion in the complex RM-U5/DN-T3 (dimer) by RNase H. The left and middle figures are a repetition of the experiment. Line 1 is a complex in water without the addition of RNase H, lines 2-9 are a complex interacting with RNase H at different time intervals. (Right) Kinetics of cleavage efficiency (proportion of digested RNA) for different experiments. The red curve corresponds to the left figure, the blue curve to the middle figure.

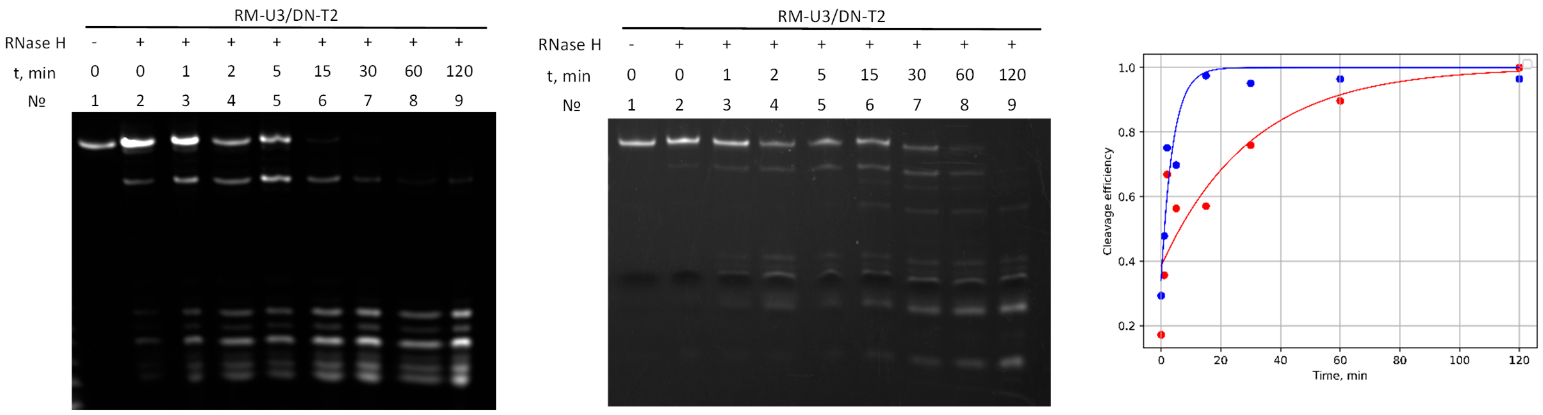

Figure S10. Electrophoretic analysis of RNA digestion in the complex RM-U3/DN-T2 (tetramer) by RNase H. The left and middle figures are a repetition of the experiment. Line 1 is a complex in water without the addition of RNase H, lines 2-9 are a complex interacting with RNase H at different time intervals. (Right) Kinetics of cleavage efficiency (proportion of digested RNA) for different experiments. The red curve corresponds to the left figure, the blue curve to the middle figure.

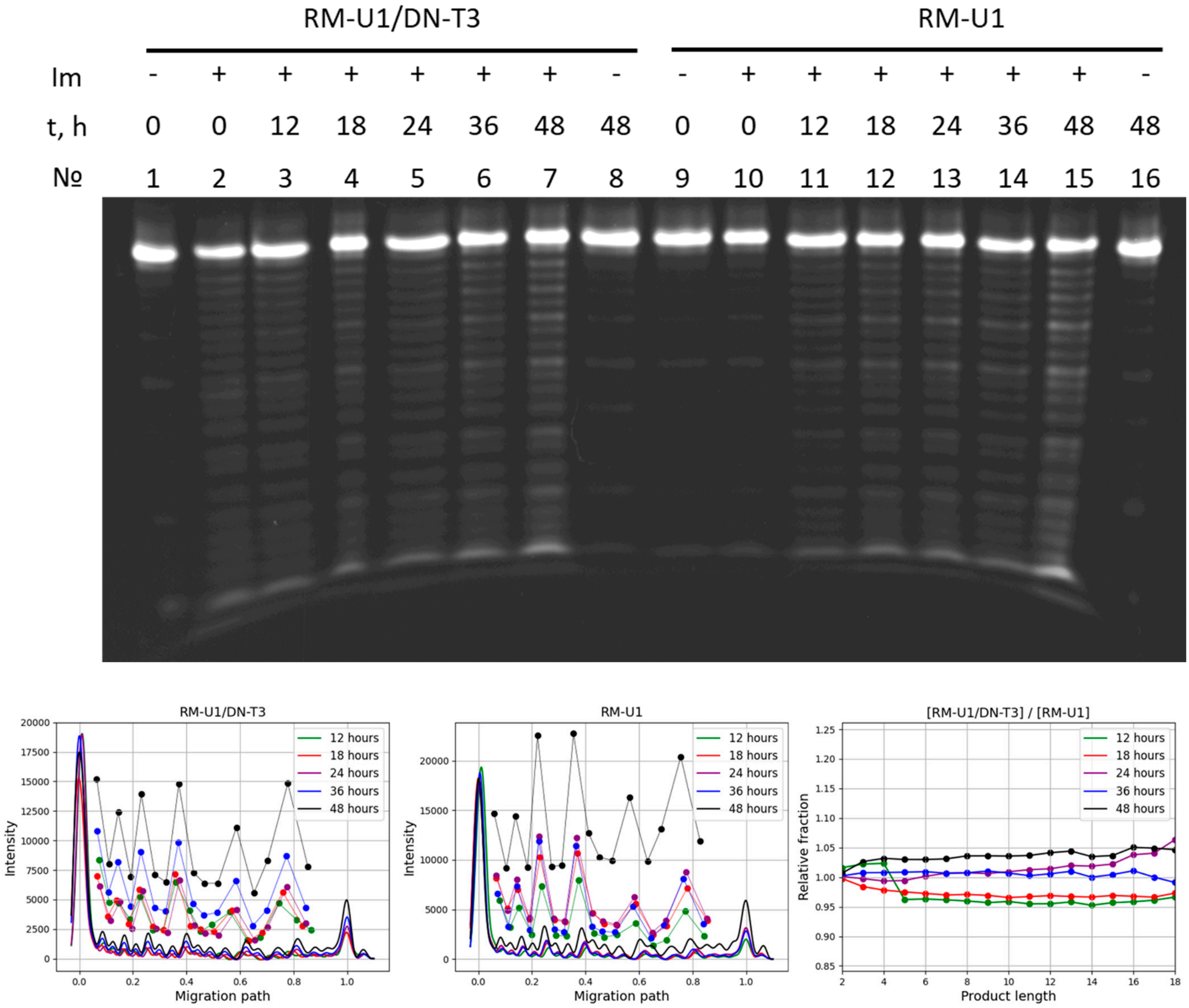

Figure S11. (Top) Electrophoretic analysis of RNA digestion in the complex RM-U1/DN-T3 (tetramer, lanes 1-8) and in the single stranded state RM-U1 (lanes 10-15) by imidazole. Lanes 1 (0 h) and 8 (48 h) are a complex in water without the addition of imidazole; lanes 2–8 and 10–15 corresponds to the different digestion times: 2–0 h, 3–12 h, 4–18 h, 5–24 h, 6–36 h, 7–48 h, 10 – 0 h, 11 – 12 h, 12 – 18 h, 13 – 24 h, 14 – 36 h, 15 – 48 h. Single-stranded RNA stability in water without the addition of imidazole: 9 – 0 h, 16 – 48 h. (Bottom) The left (RNA in the complex) and the middle (ssRNA) figures are the intensity of pixels (shown by lines) and the area under every peak in graphic's (shown by dots) at every lane. The right figure is the ratio of the areas under every peak for the RNA in the complex and in a single-stranded state.

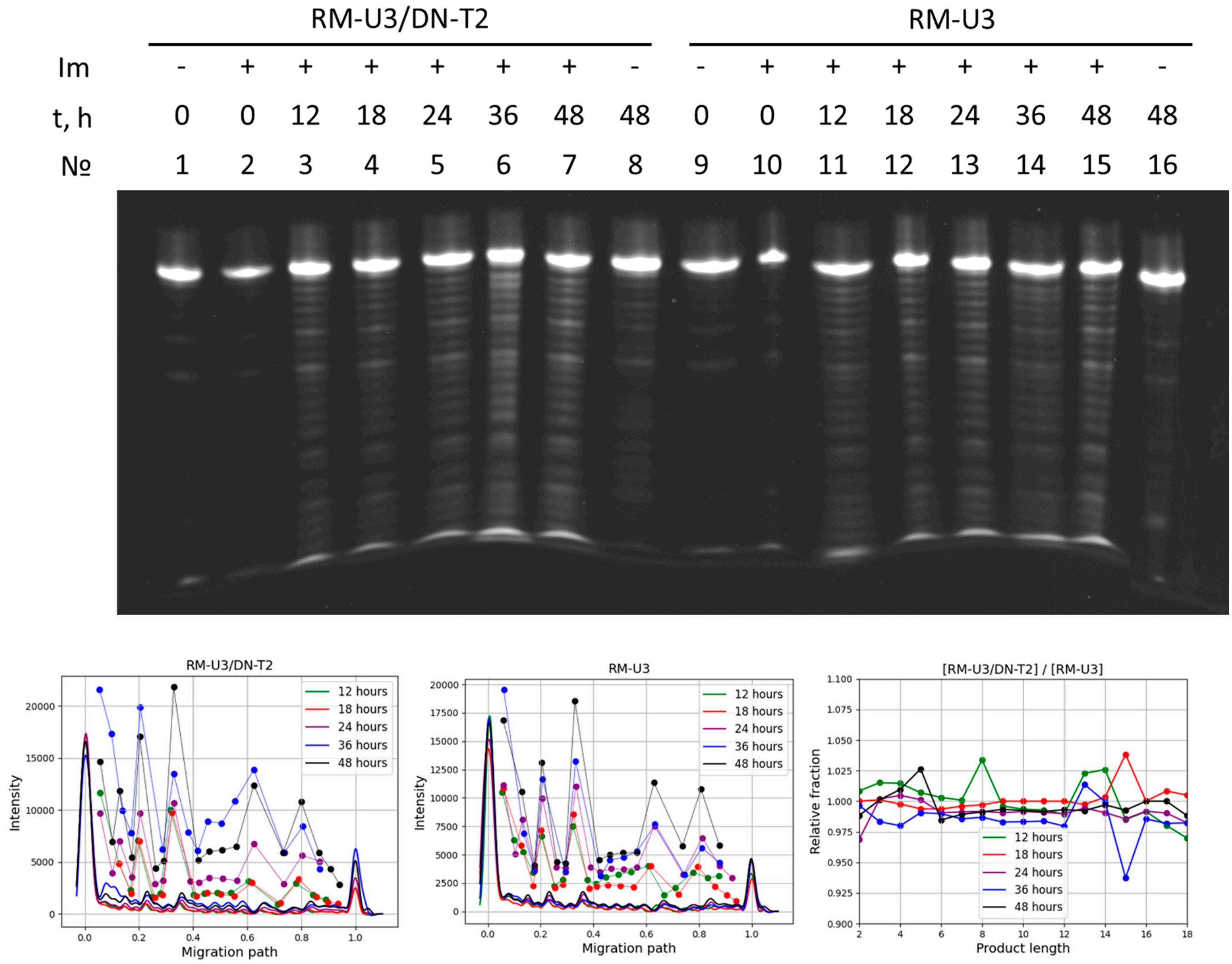

Figure S12. (Top) Electrophoretic analysis of RNA digestion in the complex RM-U3/DN-T2 (tetramer, lanes 1-8) and in the single stranded state RM-U3 (lanes 10-15) by imidazole. Lanes 1 (0 h) and 8 (48 h) are a complex in water without the addition of imidazole; lanes 2–8 and 10–15 corresponds to the different digestion times: 2–0 h, 3–12 h, 4–18 h, 5–24 h, 6–36 h, 7–48 h, 10 – 0 h, 11 – 12 h, 12 – 18 h, 13 – 24 h, 14 – 36 h, 15 – 48 h. Single-stranded RNA stability in water without the addition of imidazole: 9 – 0 h, 16 – 48 h. (Bottom) The left (RNA in the complex) and the middle (ssRNA) figures are the intensity of pixels (shown by lines) and the area under every peak in graphic's (shown by dots) at every lane. The right figure is the ratio of the areas under every peak for the RNA in the complex and in a single-stranded state.

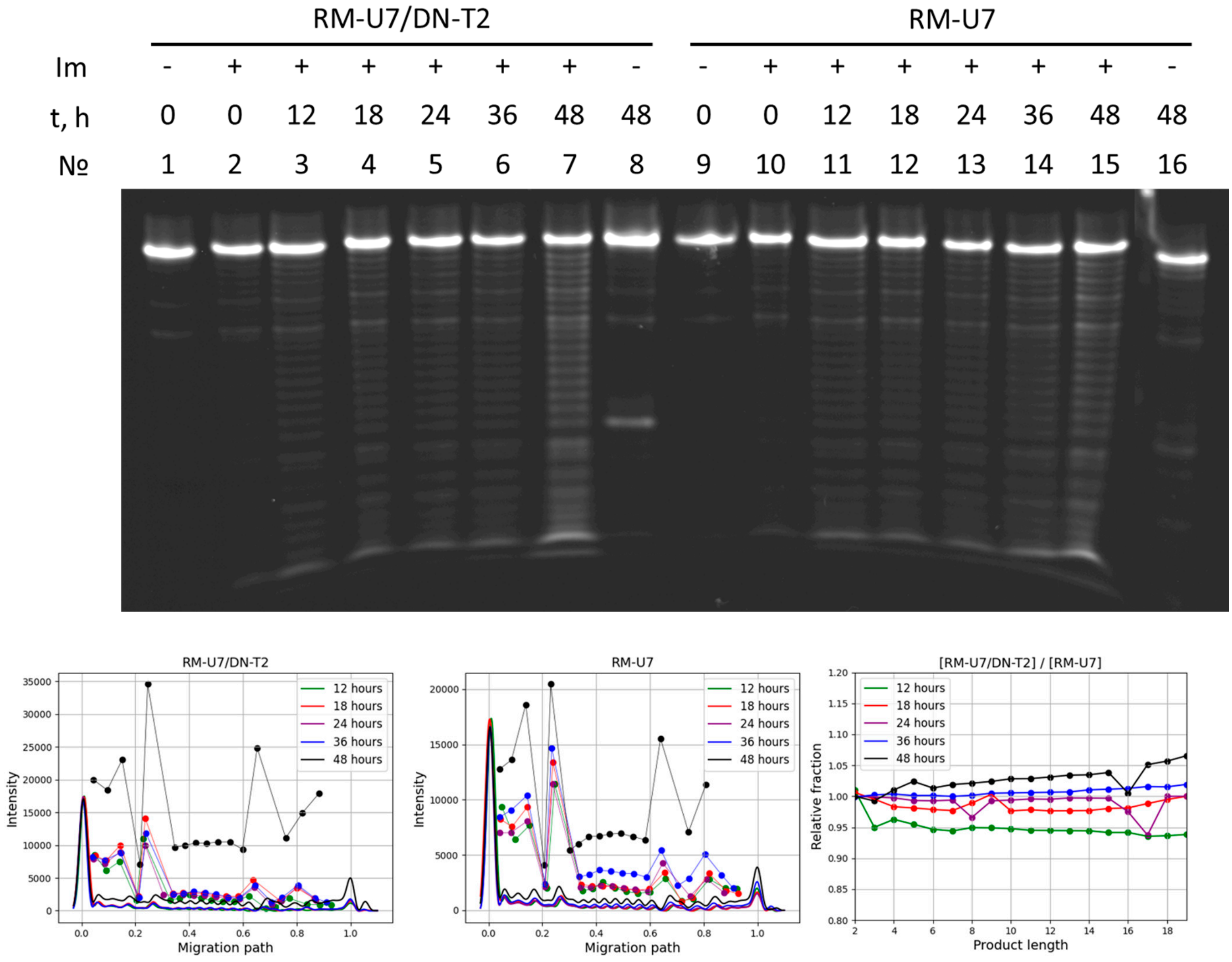

Figure S13. (Top) Electrophoretic analysis of RNA digestion in the complex RM-U7/DN-T2 (dimer, lanes 1-8) and in the single stranded state RM-U7 (lanes 10-15) by imidazole. Lanes 1 (0 h) and 8 (48 h) are a complex in water without the addition of imidazole; lanes 2–8 and 10–15 corresponds to the different digestion times: 2–0 h, 3–12 h, 4–18 h, 5–24 h, 6–36 h, 7–48 h, 10 – 0 h, 11 – 12 h, 12 – 18 h, 13 – 24 h, 14 – 36 h, 15 – 48 h. Single-stranded RNA stability in water without the addition of imidazole: 9 – 0 h, 16 – 48 h. (Bottom) The left (RNA in the complex) and the middle (ssRNA) figures are the intensity of pixels (shown by lines) and the area under every peak in graphic's (shown by dots) at every lane. The right figure is the ratio of the areas under every peak for the RNA in the complex and in a single-stranded state.

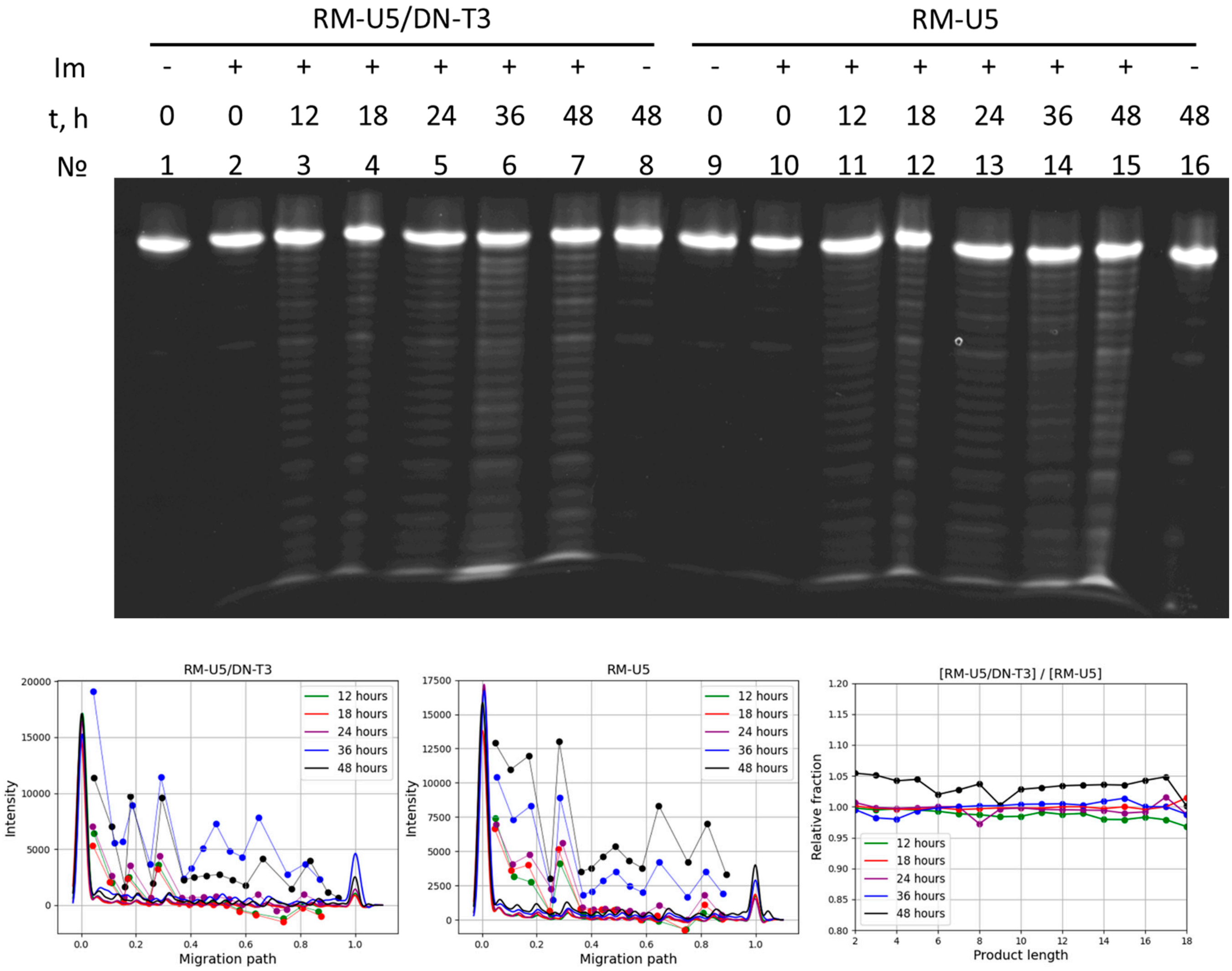

Figure S14. (Top) Electrophoretic analysis of RNA digestion in the complex RM-U5/DN-T3 (dimer, lanes 1-8) and in the single stranded state RM-U5 (lanes 10-15) by imidazole. Lanes 1 (0 h) and 8 (48 h) are a complex in water without the addition of imidazole; lanes 2–8 and 10–15 corresponds to the different digestion times: 2–0 h, 3–12 h, 4–18 h, 5–24 h, 6–36 h, 7–48 h, 10 – 0 h, 11 – 12 h, 12 – 18 h, 13 – 24 h, 14 – 36 h, 15 – 48 h. Single-stranded RNA stability in water without the addition of imidazole: 9 – 0 h, 16 – 48 h. (Bottom) The left (RNA in the complex) and the middle (ssRNA) figures are the intensity of pixels (shown by lines) and the area under every peak in graphic's (shown by dots) at every lane. The right figure is the ratio of the areas under every peak for the RNA in the complex and in a single-stranded state.

	DN	DN-T1	DN-T2	DN-T3	DN-T15	DN-T25
RM	47.8	43.5	43.6	46.0	45.6	49.4
RM-U1	41.7	38.6	36.8	40.4	42.0	43.4
RM-U2	43.7	40.4	39.0	42.1	42.9	43.0
RM-U3	40.8	38.8	37.4	41.2	42.6	41.9
RM-U5	40.7	38.4	38.2	42.0	42.7	42.5
RM-U7	40.6	39.1	39.8	42.0	42.2	42.0
RM-U10	41.0	39.0	40.8	43.2	42.6	42.2

Figure S15. Comparison of the complex type and the melting temperature.
